# The Role of Heat Shock Proteins in Type 1 Diabetes

**DOI:** 10.3389/fimmu.2020.612584

**Published:** 2021-01-14

**Authors:** Abu Saleh Md Moin, Manjula Nandakumar, Abdoulaye Diane, Mohammed Dehbi, Alexandra E. Butler

**Affiliations:** Diabetes Research Center (DRC), Qatar Biomedical Research Institute (QBRI), Hamad Bin Khalifa University (HBKU), Qatar Foundation (QF), Doha, Qatar

**Keywords:** type 1 diabetes, heat shock proteins, type 1 diabetes pathogenesis, autoantigens, metabolic stress, antigen specific immunotherapy

## Abstract

Type 1 diabetes (T1D) is a T-cell mediated autoimmune disease characterized by recognition of pancreatic β-cell proteins as self-antigens, called autoantigens (AAgs), followed by loss of pancreatic β-cells. (Pre-)proinsulin ([P]PI), glutamic acid decarboxylase (GAD), tyrosine phosphatase IA-2, and the zinc transporter ZnT8 are key molecules in T1D pathogenesis and are recognized by autoantibodies detected in routine clinical laboratory assays. However, generation of new autoantigens (neoantigens) from β-cells has also been reported, against which the autoreactive T cells show activity. Heat shock proteins (HSPs) were originally described as “cellular stress responders” for their role as chaperones that regulate the conformation and function of a large number of cellular proteins to protect the body from stress. HSPs participate in key cellular functions under both physiological and stressful conditions, including suppression of protein aggregation, assisting folding and stability of nascent and damaged proteins, translocation of proteins into cellular compartments and targeting irreversibly damaged proteins for degradation. Low HSP expression impacts many pathological conditions associated with diabetes and could play a role in diabetic complications. HSPs have beneficial effects in preventing insulin resistance and hyperglycemia in type 2 diabetes (T2D). HSPs are, however, additionally involved in antigen presentation, presenting immunogenic peptides to class I and class II major histocompatibility molecules; thus, an opportunity exists for HSPs to be employed as modulators of immunologic responses in T1D and other autoimmune disorders. In this review, we discuss the multifaceted roles of HSPs in the pathogenesis of T1D and in autoantigen-specific immune protection against T1D development.

## Introduction

Type 1 diabetes (T1D) is recognized as a condition of absolute, or near absolute, insulin deficiency due to autoimmune-mediated destruction of pancreatic β-cells and can present at any age (1). Together with low or absent plasma insulin levels, patients with T1D have elevated plasma glucagon levels and any remaining β-cells are unable to respond to insulin secretory stimuli. T1D is a catabolic condition and patients are dependent upon exogenous insulin to prevent ketosis, decrease hyperglucagonemia and normalize protein and lipid metabolism.

More than 90% of individuals with newly diagnosed T1D have at least one, and frequently multiple, autoantibodies at disease onset (2). Specific autoantibodies associated with T1D are anti-glutamic acid decarboxylase (anti-GADA), insulin autoantibodies (IAA), insulinoma-associated-2 autoantibodies (IA-2A), islet cell cytoplasmic autoantibodies (ICA) and zinc transporter 8 autoantibodies (ZnT8A) (**Table 1**).

**Table 1:** Autoantibodies associated with type 1 diabetes.

Autoantibody	Target	Specificity	References
Glutamic Acid Decarboxylase Autoantibodies [GADA]	Antibodies against an enzyme present in (but not specific to) pancreatic β-cells.	Present in 84% of patients with T1D.	(3–5)
Insulin Autoantibodies [IAA]	Antibodies targeted against the insulin molecule.	Presence of IAAs is age and sex dependent. IAAs are present in 81% of patients with TID under the age of 10 years, versus 61% in older patients. In patients <15 years, the presence of IAAs is similar in males and females; >15 years the male:female ratio is 2:1.	(3, 4)
Insulinoma-Associated-2 Autoantibodies [IA-2A]	Antibodies mounted against an enzyme present in (but not specific to) β-cells.	Present in 58% of patients with T1D.	(3, 4)
Islet Cell Cytoplasmic Autoantibodies [ICA]	Interaction between human islet cell antibodies and islet cell proteins from animal pancreas.	Present in 70–80% of new onset patients with T1D.	(3)
Zinc Transporter 8 Autoantibodies [ZnT8A]	Antibodies targeting a β-cell specific zinc transporter.	Present in 80% of patients with T1D, with 99% specificity. Provides an independent measure of autoreactivity, as 25–30% of T1D patients negative for IAA, GAD, and IA2 are ZnT8Ab positive.	(6)

The pancreas organ in T1D is decreased in weight with exocrine atrophy, lymphocytic infiltration, fibrosis and a lobular pattern of pancreatic β-cell destruction which increases with disease duration (1). Insulitis, defined as immune cell infiltrates surrounding and/or infiltrating pancreatic islets, is deemed to be the histological hallmark of T1D. However, insulitis is seen relatively infrequently in islets in human disease, likely indicating the slow progression of disease over many years. Insulitis is characterized by the infiltration of islets by macrophages, T helper cells (CD4+ or Th cells), and cytotoxic T cells (CD8+), ultimately resulting in the destruction of β-cells (7). The human leukocyte antigen (HLA) complex, representing a substantial component of the genetic risk (~50%), plays a critical role in the pathogenesis of T1D. The major histocompatibility complex encodes the human leukocyte antigen (HLA) system. Auto-antigens are presented at the β-cell surface by HLA class I molecules that are then presented to T-cells by HLA class II molecules (8). The autoimmune responses in T1D lead to a chronic β-cell inflammatory state, resulting in protein misfolding, an altered redox state in the endoplasmic reticulum (ER), ER stress and, ultimately, β-cell apoptosis. In response to ER stress, proteins like GRP78, insulin, and GAD65 undergo improper post-translational modifications that may represent neoantigens and which may therefore induce β-cell autoimmunity (9, 10).

Moreover, ER stress initiates a cellular adaptive mechanism where heat shock proteins (HSPs) play an important role, undergoing proteolytic modification and generating and presenting autoimmune antigens to class I and class II major histocompatibility complex (MHC) molecules. In this review, we will discuss what is known about the involvement of heat shock proteins (HSPs) in the pathogenesis in T1D (**Table 2**) and, conversely, the protective role played by some family members.

**Table 2:** Evidence in support of the involvement of heat shock proteins (HSPs) in the pathogenesis of T1D.

HSPs	Evidence of involvement in T1D	References
HSP60	Pancreatic β-cell surface expressed HSP60 before the appearance of islet inflammation in NOD mice.	(11, 12)
	Endogenous HSP60 (self) can act as an antigen for β-cells.	(13)
	The major autoantigenic segment of HSP60, P277, showed high specific T-cell activity in the pre-diabetic phase and at the onset of disease in children.	(14)
HSP65	The highest level of anti-HSP65 antibody was detected before the onset of diabetes in NOD mice.	(15)
	HSP65-derived fusion protein HSP65-6XP277 or His-HSP65-6IA2P2 served as vehicle for delivery of the anti-diabetogenic peptide of P277 and induced an anti-inflammatory immune response in NOD mice.	(16, 17)
HSP70	Defective HSP70 induction in response to cellular stress aggravated the inflammatory milieu against β-cells at the onset of type 1 diabetes.	(18)
	The humoral autoimmunity against HSP70, specifically, increased IgA antibody levels against HSP70 protein, was also observed in humans with T1D.	(19)
	HSP70 bound to the immunogenic fragment of proinsulin facilitated the interaction with antigen presenting cells (APCs) *via* different cell surface receptors.	(20–22)
HSP90	Elevated levels of circulating IgG1 and IgG3 class-switched anti-Hsp90 autoantibodies have been consistently identified in individuals in the latent or pre-clinical stages of T1D.	(23)
DNAJ family	Higher HSP40-creatinine ratio was observed in urine in patients with T1D.	(24)
HSP10	In humans, HSP10 autoantibodies have been detected in a high proportion of the patients with newly diagnosed fulminant type 1 diabetes (FT1DM) and acute onset type 1 diabetes (AT1DM).	(25)
HSP27	Independent association between serum HSP27 (sHSP27) and distal symmetrical polyneuropathy (DSP) in patients with T1D has been documented.	(26)

## Metabolic Stress and Generation of Autoantigens in T1D

Human and animal studies have revealed that metabolic stress promotes protein misfolding and/or post-translational aberrations, β-cell dysfunction and, ultimately, apoptosis. It is now accepted that immune cells preferentially recognize a subset of post-translationally modified peptides and misfolded proteins from β-cells, known as neoautoantigens, that are capable of generating an autoimmune response and thus, contribute to β-cell destruction (27). The upregulation of key ER stress markers has been documented both in islets of humans with T1D and in Non-Obese Diabetic (NOD) mice (28, 29). In response to ER stress, for example, insulin and GAD65 proteins undergo inappropriate post-translational modification and/or folding and the modified protein products are believed to act as immunogenic neoautoantigens that could be presented by the MHC to induce β-cell autoimmunity (9, 10). In support of this, mitigation of ER stress with chemical chaperones prevents the development of T1D in NOD mice (30).

Likewise, chronic inflammation may impair insulin secretion by β-cells and/or lead to the formation of neoautoantigens that may trigger islet autoimmunity (31). Furthermore, the array of inflammatory mediators, such as IL-1β, TNF-α and IFN-γ, negatively impacts the function and survival of β-cells, and can promote β-cell apoptosis (32).

Excessive oxidative stress resulting from perturbation of redox homeostasis plays a critical role in the pathophysiology of T1D (33). This response is induced when the host scavenging system is overwhelmed with the accumulation of free radicals, such as reactive oxygen (ROS) and reactive nitrogen species (RNS) (34). The reduced levels of endogenous antioxidant defense system components in β-cells makes them highly vulnerable to those reactive by-products as well as to proinflammatory cytokines (35). Impairment of redox homeostasis has been implicated in the impairment of β-cell function (36). In addition, ROS act on the innate immune system to induce the production of inflammatory cytokines, such as TNF-α and IL-1β, that ultimately lead to the activation of CD4+ and CD8+ T cells (33, 37). Consistent with this, overexpression of antioxidant enzymes has been shown to protect insulin-producing cells from the damage caused by both oxidative stress and inflammatory cytokines (38). Furthermore, mimetics of the antioxidant enzymes and antioxidant molecules, such as quercetin, are effective in preserving β-cell mass and protecting animals from development of T1D (39, 40). Taken together, these pieces of evidence suggest that metabolic stress is involved in the onset of T1D by impairing β-cell function and promoting β-cell destruction.

## Cellular Metabolic Stress Responders: Heat Shock Proteins (HSPs) in the Pathogenesis of T1D

The cellular stress response is a self-protective mechanism that counteracts environmental stresses and is mediated by a group of evolutionally conserved proteins, the heat shock proteins (HSPs) (41). The HSPs were originally described for their role as chaperones that regulate the conformation and function of a large number of cellular proteins in order to protect the body from stress. HSPs are, however, additionally involved in facilitating tumor antigen uptake and processing through MHC Class I and class II pathways in antigen presenting cells (for example, in dendritic cells) (42–44); thus, this may represent an opportunity for HSPs to be used to modulate immunologic responses in T1D as well as other autoimmune disorders. In this section, we will discuss the multifaceted roles of heat shock proteins (HSPs) in T1D (**Table 2**).

## HSP60

Heat shock protein 60 (HSP60) is one of the most intensely studied HSPs, especially in relation to a number of autoimmune and inflammatory diseases such as rheumatoid and juvenile idiopathic arthritis, atherosclerosis, juvenile dermatomyositis and diabetes (45–47). HSP60 is normally expressed in mitochondria, where it assists in the folding of small and soluble proteins in the mitochondrial matrix (48, 49). Mitochondrial stress conditions cause upregulation of HSP60 (48).

HSP60 demonstrates a direct link between innate immunity and autoimmunity in the pancreatic islets, since β-cells of NOD mice show expression of HSP60-related proteins (11) on their surface before the appearance of islet inflammation (12). Autoantibodies against self-HSP60 were also found to be associated with various autoimmune diseases such as T1D (50). HSP60 is a highly conserved protein, therefore both bacterial (foreign) and endogenous (self) HSP60 can act as an antigen for β-cells (13). However, the tissue specificity of HSP60, especially how and why β-cells are the preferred target for the HSP60-mediated T-cell response, is not fully understood. It has been proposed that systemic tissue-specific triggers such as IFN-γ, consequent upon a viral infection, may augment β-cells to become targets for anti-HSP60 T-cells (51). Moreover, it has also been suggested that HSP60 resides in a unique way in the secretory vesicle of the β-cell and, in the process of insulin secretion, these vesicles fuse to the β-cell membrane resulting in exposure of HSP60 to the extracellular environment even in the absence of mitochondrial stress (51, 52). Previous studies have shown HSP60-induced vascular endothelial cell damage *via* the toll-like receptor-4 (TLR-4)-activated NF-κB pathway (53). The pathogenic mechanism of HSP60 in T1D is mediated by the hyperproduction of the proinflammatory mediator IL-12 (p70) in macrophages of diabetes-prone NOD mice (54).

**Figure 1:**
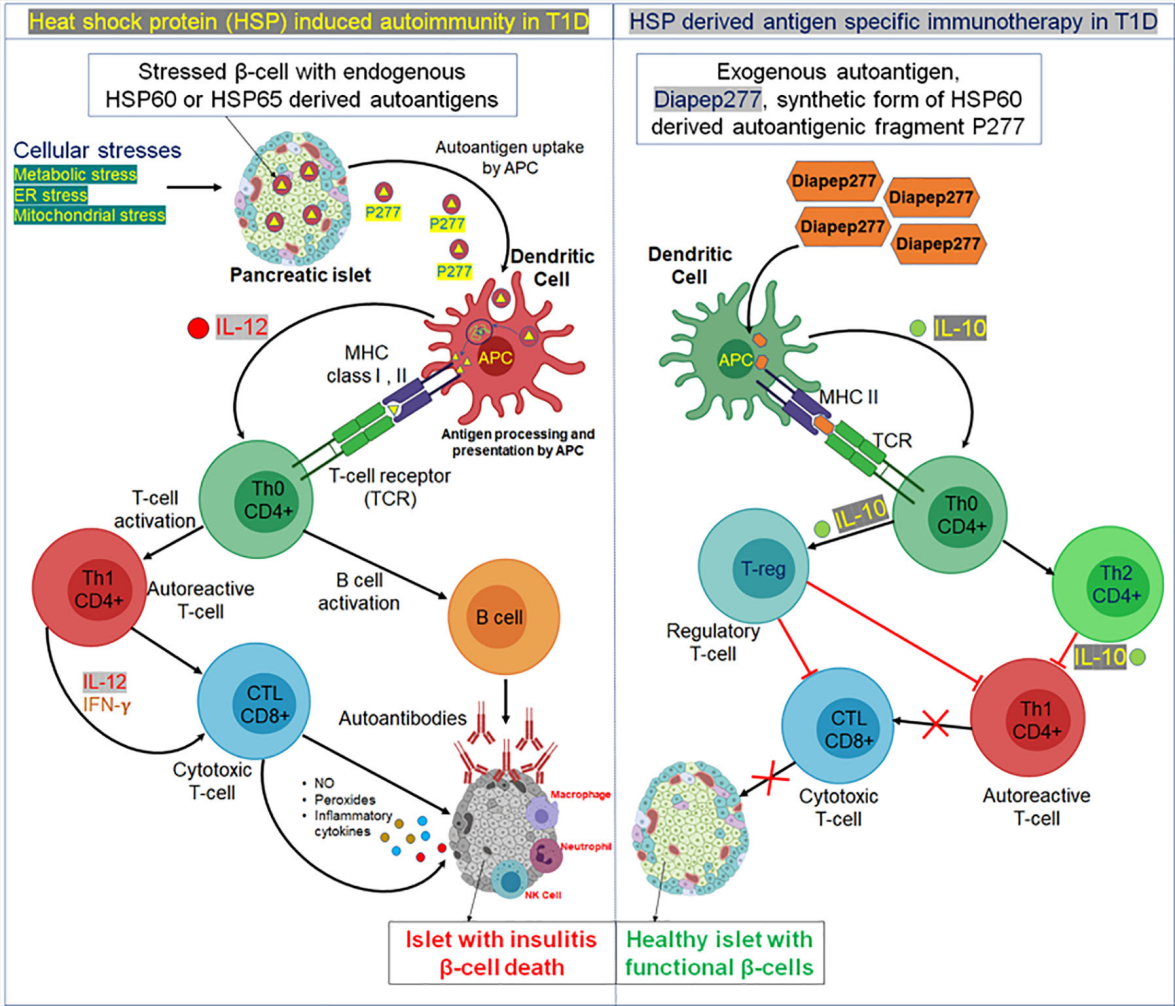
Possible mechanism of heat shock protein (HSP)-induced autoimmunity and HSP-derived antigen immunotherapy in T1D. *Left panel*, in response to cellular stresses, like ER stress or mitochondrial stress, HSPs undergo improper post-translational modifications and generate autoantigens. For example, in response to cellular stress, an autoreactive peptide fragment P277 is produced from HSP60 or HSP65 and released with the insulin granules from a pancreatic β-cell. The secreted P277 fragment is then taken up by antigen presenting cells, specifically dendritic cells and processed in lysosomal vesicles. Transfer of peptide fragments of the autoantigen to MHC class I or II molecules then occurs, that migrate to the plasma membrane and cross-present the autoantigen fragments (either by MHCI or MHCII) to cognate T cell receptors on naïve T helper cells (Th0). At the same time, dendritic cell processing of the autoantigen stimulates biosynthesis and secretion of the inflammatory cytokine interleukin 12 (IL-12) which stimulates the Th0 cells to undergo morphogenesis into autoreactive effector T helper cells (Th1). The autoreactive Th1 cells secrete inflammatory cytokines, such as IFN-γ and IL-2; this then stimulates cytotoxic lymphocytes (CTL) to secrete nitric acid, peroxide, and several other inflammatory cytokines that stimulate pancreatic islet inflammation (insulitis). Th0 cells also activate B cells to produce autoantibodies against β-cells expressing HSP autoantigen 277. This hyperinflammatory reaction is enhanced by migration of other immune cells like macrophages, neutrophils, and natural killer (NK) cells to the pancreatic islet. Thus, the chronic insulitis results in ongoing pancreatic β-cell death resulting in increasing insulin deficiency and a progressive increase in blood sugar (hyperglycemia). *Right Panel*, Exogenous administration (oral or subcutaneous delivery) of small amounts of synthetic fragments of the HSP60/HSP65-derived islet autoantigen, Diapep277, that exerts a protective antigen-specific therapeutic effect by stimulating dendritic cells. Upon administration, Diapep277 is taken up by dendritic cells, processed and presented by MHCII molecules to naïve Th0 cells. Activated dendritic cells also secrete the anti-inflammatory cytokine IL-10 which stimulates naïve cognate Th0 lymphocytes to undergo morphogenesis into anti-inflammatory CD4+ Th2 helper cells that in turn secrete IL-10 which suppresses further development of autoreactive Th1 cells and decreases potential insulitis onset. Alternatively, naive Th0 cells may develop into one of several subclasses of regulatory T cells (Treg), which can block Th1 and CTL development leading to prevention of pro-inflammatory cytokine-induced insulitis of pancreatic islets resulting in retention of functional β-cells in T1D.

While HSP60 activates macrophages and dendritic cells by promoting proinflammatory effectors, the major autoantigenic segment of HSP60 is P277, a 24-residue peptide (VLGGGCALLRCIPALDSLTPANED) in the C-terminal of the HSP60 protein, that promotes anti-inflammatory cytokine production to regulate immune responses *via* toll-like receptor-2 (TLR-2) (**Figure 1**) (55, 56). A study performed on serum samples from children with T1D demonstrated that P277 peptide-specific T-cell activity was high in the pre-diabetic phase and, at the onset of disease, decreased markedly (10). Treatment of NOD mice with P277 peptide enhanced the survival of residual β-cell function even late in the course of autoimmunity, after the onset of clinical hyperglycemia (57). The protective effect of P277 peptide resulted from a shift in the cytokine profile of HSP60 autoimmunity from a proinflammatory Th1 phenotype to anti-inflammatory Th2 phenotype (58, 59). This suggests that HSP60 and HSP60-derived peptides can induce both proinflammatory and anti-inflammatory cytokines, confirming HSP60 as an important modulator of inflammation in T1D mellitus. Thus, the beneficial effect of P277 in controlling the inflammatory response against β-cells in T1D opens up a new therapeutic opportunity.

DiaPep277, the synthetic peptide derived from HSP60, functions as an epitope of HSP60 and showed the highest anti-HSP60 T-cell response (60). Oral administration of DiaPep277 increased IL-4 and IL-10 secretion and decreased IFN-γ secretion, meaning that it induced a Th2 response which reduced Th1 cell-induced autoimmunity to HSP60, GAD, and insulin; the T-cell response to bacterial antigen peptide treatment in DiaPep277 treated mice was, however, unaffected (58, 61). A randomized, double-blind clinical trial with DiaPep277 peptide treatment in patients with recent onset T1D has shown promising responses. DiaPep277 appeared to preserve endogenous insulin production through induction of a shift from Th1 to Th2 cytokine production by the autoimmune T-cells (62) (**Figure 1**). However, other similar studies carried out in children with recent onset T1D showed no beneficial effect in preserving β-cell function or improving metabolic control (63–65). Another randomized double-blind phase I/II clinical trial of P277 peptide therapy reported that, when administered to C-peptide positive patients, preservation of β-cell function resulted; mechanistically, this was due to anti-inflammatory IL10 production prevailing over therapy-generated cytokines, and no serious adverse effects were reported (66, 67).

## HSP65

Mycobacterium tuberculosis heat shock protein 65 (HSP65) is another well-known member of the HSP family; it contains numerous B and T-cell epitopes and can thus evoke a strong T-cell-dependent immune response (16, 17, 68). HSP65 shares 50% homology with human HSP60 (69); therefore, upon stimulation with bacterial HSP65, a cross immune response to host HSP60 is likely. Studies in NOD mice have shown the highest level of anti-HSP65 antibody detection before the onset of diabetes (15). The strong association of onset of β-cell destruction with development of anti-HSP65 T-lymphocytes in NOD mice (70) also suggests that the reaction to HSP65 may confer greater susceptibility to T1D in humans (71). Serological immunity, meaning IgG antibody cross-reactivity with mycobacterial HSP65, has also been shown to occur in humans with T1D (72). Thus, the unique “molecular mimicking” characteristic of HSP65 could serve as an advantageous carrier for a peptide-based vaccine, one therapeutic approach for the treatment of T1D. Nasal administration of the fusion protein HSP65-6 X P277 served as a vehicle for delivery of the peptide P277 and induced an anti-inflammatory immune response in NOD mice (16). Another HSP65-P277 fusion protein, His-HSP65-6IA2P2, delivered to NOD mice through nasal immunization also prevented development of diabetes (73), suggesting that HSP65 is a key modulator in resetting the immunological paradigm to prevent β-cell-specific inflammation.

## HSP70

The HSP70 superfamily (ranging in size from 66 to 78 KDa), is a group of cytosolic ATP-dependent chaperones that also constitute a group of “autoantigens” with the potential to trigger immunoregulatory pathways in human inflammatory diseases such as rheumatoid arthritis (RA), T1D, and atherosclerosis (74, 75). Previous studies have demonstrated the role of HSP70 in induction of β-cell directed immunity and in the development of insulin-deficient diabetes (76). HSP70 expression is increased in response to cellular stress, resulting in efficient protection against β-cell damaging mediators in isolated rat islets and in human β-cells (77, 78). HSP70 also plays a cyto-protective role in selenium nanoparticle (SeNP)-mediated prevention of progression of type 1 diabetic nephropathy (79). A deficit in stress-induced HSP70 expression in islets of an animal model of human T1D [BioBreeding diabetes-prone (BB-DP) rats] suggests a protective role for HSP70 in T1D (80, 81). The protective role of HSP70 also involves regulating metabolic pathways in T1D. HSP70 mediated heat-preconditioning attenuated streptozotocin (STZ)-induced metabolic alterations in hepatic carbohydrate metabolism and the oxidative state in T1D model rats (82). Defective HSP70 induction in response to cellular stress also aggravated the inflammatory milieu against β-cells at the onset of T1D (18). The humoral autoimmunity against HSP70 protein (specifically, increased IgA antibody levels against HSP70) was also observed in humans with T1D (19).

The direct immunogenic property of any particular peptide fragment of HSP70 has not yet been elucidated. However, its role in the possible contribution of another peptide recognition system to the selection of amino acid stretches for presentation by MHC molecules to T-cells has been well studied. HSP70 and other cytosolic chaperones have been found to bind peptides from antigenic proteins and to deliver such peptides to MHC class I or class II molecules, respectively (83). HSP70 binds to selected regions of the insulin molecule, specifically to a central hydrophobic leucine-rich core flanked by regions enriched for basic amino acids. These regions are superimposed upon the major target regions of MHC class II restricted T-cell autoimmunity to proinsulin in T1D and NOD mice (84). The interaction of HSP70 and proinsulin may occur within β-cells or in the extracellular environment, since HSP70 may also be incorporated in the secretory vesicle granules during proinsulin synthesis and released into the extracellular space (85). Upon binding of HSP70 to the immunogenic fragment of proinsulin, antigen presenting cells (APCs) can take up HSP70 or HSP70-peptide complexes *via* several cell surface receptors including CD91, LOX-1, and the Siglec family (20–22). In addition, immunogenic proinsulin peptide fragments can also be transferred to neighboring macrophages or dendritic cells through secretory vesicles or exosomes which also contain HSP70 (86–88). Thus, the peptide sequence positioned at the peptide binding cleft of HSP70 is not easily accessible to proteolytic cleavage but is available for transfer to MHC class II molecules for antigen presentation to T-cells to induce autoimmunity against β-cells in T1D.

## HSP90

HSP90 is a highly conserved member of the heat shock protein family of molecular chaperones which is involved in numerous cellular processes including innate and adaptive immunity (89). Elevated levels of HSP90 were observed in islets of NOD mice prior to the onset of hyperglycemia (90). Elevated levels of circulating IgG1 and IgG3 class-switched anti-HSP90 autoantibodies have been consistently identified in individuals in the latent or pre-clinical stages of T1D, suggesting HSP90 as a potential biomarker for early detection of T1D (23). However, HSP90 may not be a marker of T1D disease progression in human. A recent study using human serum samples from the TrialNet Pathway to Prevention (PTP) study demonstrated that HSP90 levels were not different between autoantibody positive T1D progressors and non-progressors (91).

Whether HSP90 is protective or detrimental in T1D is not fully understood; however, a recent study in a T1D mouse model suggested that HSP90 might play a dual role in T1D (92). HSP90 is involved in regulating MHC class II presentation of both exogenous and endogenous GAD antigen by human B cells (93). Since GAD is also an autoantigen for T1D, it is likely that HSP90 may also participate in antigen processing and presentation of autoantigen in pancreatic β-cells in T1D. The mechanism by which HSP90 modulates antigen presentation is not fully understood; however, in NOD mice, it has been shown that HSP90 is secreted extracellularly in exosomes (94), suggesting a receptor-mediated uptake of HSP90 immunogenic peptide fragments by APCs to induce T cell-mediated autoimmunity in T1D.

## DNAJ Family Proteins

The involvement of DNAJ family proteins in T1D has not been extensively studied to date. However, a large meta-analysis of genome wide genotyped datasets for T1D revealed that DnaJ/HSP40 homolog, subfamily C, member 27 gene (DNAJC27) is associated with T1D (95). A higher HSP40-creatinine ratio was observed in the urine of patients with T1D, suggesting a strong association of DNAJ family proteins in progressive renal injury in T1D (24). Mutations leading to loss of another ER-DNAJ family protein, DNAJC3, have been shown to result in pancreatic β-cell failure and diabetes in mice (96). Consistent with the animal findings, pedigree analysis and whole exome sequencing in two index families also revealed that a loss of function *DNAJC3* mutation (resulting in absence of DNAJC3 protein in ER) manifested as monogenic diabetes and multisystemic neurodegeneration (97).

In contrast to these observations, a recent study demonstrated that DNAJC3 facilitated the cytokine-induced translocation and secretion of the ER chaperone glucose-regulated protein 78 (GRP78; also known as BiP) in rodent and human β-cells (98). This, in turn, may serve as an autoantigen of β-cells, as secreted GRP78 may have immunogenic characteristics against which the generation of autoantibodies has been reported (99). Therefore, DNAJ family proteins may play a dual role in the pathogenesis of T1D, and further studies are needed to fully elucidate the underlying mechanisms.

## Other HSPs (HSP10 and HSP27)

HSP10 is a 10 kDa, highly conserved, mitochondrion-resident protein, possessing anti-autoimmunity properties. In a murine model of experimental autoimmune encephalomyelitis (EAE), recombinant HSP10 played a major role in reduction of phenotypic disease (100). In humans, HSP10 autoantibodies have been detected in a high proportion of patients with newly diagnosed fulminant T1D (FT1DM) and acute onset T1D (AT1DM), suggesting that autoantibodies to HSP10 are new diagnostic and clinical markers of T1D (25). Another highly conserved heat shock protein, HSP27, acts as a filament stabilizer under stress conditions, and has also been found to be associated with microvascular complications in T1D. The cross-sectional, nested, case-control study from the EURODIAB Prospective Complications Study in patients with T1D revealed an independent association between serum HSP27 (sHSP27) and distal symmetrical polyneuropathy (DSP), suggesting sHSP27 levels may be a novel biomarker of diabetic neuropathy (26).

## Translational Aspects of HSP Studies in Type 1 Diabetes

Given the important roles that heat shock proteins play in normal and pathological conditions, their potential as therapeutic agents or targets for T1D has recently gained attention. DiaPep277, the synthetic peptide of the HSP60-derived peptide p277, has been shown to be effective as a modulator of the immune system by means of autoantigen vaccination as well as by modulating the innate immune system to preserve β-cell reserve in T1D. In vitro measurement of T-cell reactivity to Diapep277 demonstrated that, compared with the placebo-treated group, patients treated with Diapep277 produced less IFNγ and more IL-10 and IL-13 in response to the p277 peptide, indicating an enhanced TH2 cytokine phenotype (101). DiaPep277 also acts as a co-stimulator of human regulatory T cells. The signal transduction cascade induced by the p277 peptide involves suppression of cytokine signaling 3 (SOCS3) expression and signal transducer and activator of transcription 3 (STAT3) activation and other identified downstream targets (101). Administration of DiaPep277 protected NOD mice against insulitis (57) and delayed the onset of diabetes in BB-DP rats (102), suggesting Diapep277 to be a promising candidate for a T1D vaccine. Interestingly, treatment with DiaPep277 in pre-clinical animal models of diabetes also modified the TH1 responses to other β-cell and diabetes-related autoantigens, such as insulin and GAD (58, 103). Clinical trials with DiaPep277 have also shown promising results. DiaPep277 was reported immunologically safe in a clinical trial as the majority of subjects demonstrated a peptide-specific anti-inflammatory cytokine profile dominated by production of the regulatory cytokine IL-10 (66). Another clinical study showed significant preservation of stimulated C-peptide production for up to 18 months in response to 1.0 mg of DiaPep277 in patients with T1D (62).

In addition to immunomodulation, HSPs may be viewed as survival proteins as well as disease biomarkers, possessing an intrinsic ability to confer protection against diabetic micro and macrovascular complications. In a large cohort of patients with T1D enrolled in the EURODIAB PCS study, higher circulating HSP27 levels were found to confer a twofold increased risk of distal symmetric polyneuropathy (DSP), independent of known risk factors and confounders (26). HSP70 may be beneficial in early diabetic nephropathy (DN) because of its intracellular cytoprotective activity. However, a small study performed in diabetic patients showed an association between urinary HSP70 levels and albuminuria (104). Moreover, serum HSP70 levels were higher in diabetic patients with albuminuria (105).

Several naturally occurring xenohormetic compounds (bioactive compounds that are produced in response to cellular stress) have been identified (106), which increase the expression of HSPs and consequently have therapeutic benefits in diabetes. For example, Alphalipoic acid (ALA), a potent natural antioxidant and an essential co-factor of mitochondrial enzymes has been shown to improve glucose uptake and has proven therapeutic benefits in diabetes and its associated complications such as neuropathy, nephropathy, and hypertension (107). Administration of ALA increased expression of HSF-1 and HSP70 in the kidney of streptozotocin-induced diabetic (SID) rats and protected them from their natural progression to nephropathy (108). HSP90 promotes cell survival, migration, inflammation, and angiogenesis, and is therefore considered to be a very promising target in cancer therapy. This has led to development of specific HSP90 inhibitors that are currently undergoing clinical testing in humans as chemotherapeutics. Recently, these compounds have also been tested in diabetic animals, revealing the benefit of HSP90 blockade in diabetic complications. For example, treatment of diabetic *db/db* mice on a high fat diet (HFD) with 17-Dimethylaminoethylamino-17-demethoxygeldanamycin (17-DMAG) (the earliest and best characterized HSP90 inhibitor) preserved kidney function and ameliorated glomerular and tubular damage induced by HFD (109). HSP90 may also be beneficial in wound healing in T1D. A fragment of secreted HSP90, Hsp90α(F-5) accelerated diabetic wound healing in mice (110).

## Limitations of the HSP Studies in Diabetes Research

The research to date on HSPs in the field of diabetes has limitations. A study reporting T-cell proliferative responses and HSP60-derived epitope-specific cytokine production should be interpreted with caution as patient numbers were small (111). Genetic polymorphism in HSP genes also present a confounding factor for interpretation of both pre-clinical and clinical data in HSPs-T1D studies. For example, the genes that encode for inducible HSP70 in human, rat and mouse are located with genes of the major histocompatibility complex (MHC) (112–114), and there is a well-recognized strong association between certain MHC genes and T1D susceptibility (115). Consequently, differences in HSP70 genotypes were found between control and T1D patients (116), and the role HSP70 plays in relation to T1D onset requires clarification. Another key limitation is the difficulty of interpreting whether changes in HSP expression in target organs with diabetic complications indicates direct involvement of HSPs in the pathogenic process or a compensatory cytoprotective response at different stages of the disease. Moreover, the opposing effects of intra and extracellular HSPs often leads to conflicting conclusions being drawn from HSP protein expression studies. For example, intracellular HSP70 is cytoprotective in defending renal cells exposed to the diabetic milieu, while extracellular HSP70 (eHSP70) causes tubulo-interstitial damage (117). This “dual-response” of a single HSP in different conditions limits their use as therapeutic options in T1D. In addition, pharmacological modulation of one HSP can enhance the expression of other HSPs which, in turn, can oppose its action. One example is inhibition of HSP90 by Celastrol (a Chinese traditional anti-inflammatory medicine) that induced a robust increase in critical heat shock proteins such as HSP70, HSP27, and HSP32 in neuronal cells (118). Considering such evidence, it is apparent that not all HSPs will be suitable therapeutic targets for T1D prevention. Therefore, intervention studies targeting specifically either intra or extracellular HSPs are required to gain deeper insight into the functional role of HSPs in T1D and its related complications.

## Conclusions and Future Perspectives

The occurrence of T1D is increasing worldwide. Despite better insulins, and progress in islet transplantation and other techniques to facilitate treatment, the disease is still very serious, with a high risk of complications (119). HSPs have been considered as the immunodominant antigens of insulitis in T1D. Autoantibodies as well as effector pathological T cells reactive to self HSPs (for example, T cells reactive to HSP60) were found in T1D, suggesting a close association of HSPs in the pathogenesis of T1D. Interestingly, HSPs play a dual role in T1D: apart from serving as danger signals to pancreatic β-cells, peptides derived from HSPs also act as an inducers of regulatory T cell responses that can dampen inflammation in T1D. Given the complexity of the immune response targeting insulin-producing β-cells, it is unlikely that a single therapy could represent an optimally effective way to treat T1D; hence, autoantigen treatment in combination therapies may be a way forward. HSP-based antigen specific therapies hold great promise for preventing or inhibiting β-cell destruction in T1D. HSPs and their auto-antigenic peptide fragments are able to stimulate a T-cell response and thus can restore tolerance of β-cells against autoantigens. Moreover, HSPs can potentially be used as biomarkers for those at risk prior to the onset of T1D. Taken together, targeting HSPs may represent a novel way to circumvent the obstacles of current therapeutic strategies in the treatment and prevention of T1D.

## Author Contributions

AM, MN, AD, MD, and AB wrote the manuscript. MD and AB conceived the idea for the project. AB is the guarantor of this work. All authors gave their consent for publication. All authors contributed to the article and approved the submitted version.

## Conflict of Interest

The authors declare that the research was conducted in the absence of any commercial or financial relationships that could be construed as a potential conflict of interest.

## References

[1] Atkinson MA Eisenbarth GS Michels AW Type 1 diabetes. Lancet (2014) 383(9911):69–82. 10.1016/S0140-6736(13)60591-7 23890997 PMC4380133

[2] Ziegler AG Nepom GT Prediction and pathogenesis in type 1 diabetes. Immunity (2010) 32(4):468–78. 10.1016/j.immuni.2010.03.018 PMC2861716 20412757

[3] Tsirogianni A Pipi E Soufleros K Specificity of islet cell autoantibodies and coexistence with other organ specific autoantibodies in type 1 diabetes mellitus. Autoimmun Rev (2009) 8(8):687–91. 10.1016/j.autrev.2009.02.019 19217947

[4] Bingley PJ Bonifacio E Mueller PW Diabetes Antibody Standardization Program: first assay proficiency evaluation. Diabetes (2003) 52(5):1128–36. 10.2337/diabetes.52.5.1128 12716742

[5] Vives-Pi M Somoza N Vargas F Armengol P Sarri Y Wu JY Expression of glutamic acid decarboxylase (GAD) in the alpha, beta and delta cells of normal and diabetic pancreas: implications for the pathogenesis of type I diabetes. Clin Exp Immunol (1993) 92(3):391–6. 10.1111/j.1365-2249.1993.tb03410.x PMC1554778 8513574

[6] Wenzlau JM Frisch LM Gardner TJ Sarkar S Hutton JC Davidson HW Novel antigens in type 1 diabetes: the importance of ZnT8. Curr Diabetes Rep (2009) 9(2):105–12. 10.1007/s11892-009-0019-4 19323954

[7] Foulis AK McGill M Farquharson MA Insulitis in type 1 (insulin-dependent) diabetes mellitus in man–macrophages, lymphocytes, and interferon-gamma containing cells. J Pathol (1991) 165(2):97–103. 10.1002/path.1711650203 1744803

[8] Trowsdale J Knight JC Major histocompatibility complex genomics and human disease. Annu Rev Genomics Hum Genet (2013) 14:301–23. 10.1146/annurev-genom-091212-153455 PMC4426292 23875801

[9] Sherr J Sosenko J Skyler JS Herold KC Prevention of type 1 diabetes: the time has come. Nat Clin Pract Endocrinol Metab (2008) 4(6):334–43. 10.1038/ncpendmet0832 18446141

[10] Mannering SI Harrison LC Williamson NA Morris JS Thearle DJ Jensen KP The insulin A-chain epitope recognized by human T cells is posttranslationally modified. J Exp Med (2005) 202(9):1191–7. 10.1084/jem.20051251 PMC2213236 16260488

[11] Brudzynski K Cunningham IA Martinez V A family of hsp60-related proteins in pancreatic beta cells of non-obese diabetic (NOD) mice. J Autoimmun (1995) 8(6):859–74. 10.1016/S0896-8411(95)80022-0 8824711

[12] Brudzynski K Insulitis-caused redistribution of heat-shock protein HSP60 inside beta-cells correlates with induction of HSP60 autoantibodies. Diabetes (1993) 42(6):908–13. 10.2337/diabetes.42.6.908 8098696

[13] Kaufmann SH Immunity to bacteria. Curr Opin Immunol (1989) 2(3):353–9. 10.1016/0952-7915(89)90141-6 2534501

[14] Horváth L Cervenak L Oroszlán M Prohászka Z Uray K Hudecz F Antibodies against different epitopes of heat-shock protein 60 in children with type 1 diabetes mellitus. Immunol Lett (2002) 80(3):155–62. 10.1016/S0165-2478(01)00336-4 11803047

[15] UK Prospective Diabetes Study 6 Complications in newly diagnosed type 2 diabetic patients and their association with different clinical and biochemical risk factors. Diabetes Res (1990) 13(1):1–11. 2097090

[16] Liang J Aihua Z Yu W Yong L Jingjing L HSP65 serves as an immunogenic carrier for a diabetogenic peptide P277 inducing anti-inflammatory immune response in NOD mice by nasal administration. Vaccine (2010) 28(19):3312–7. 10.1016/j.vaccine.2010.02.100 20226247

[17] Jin L Wang Y Xiong Q Chen Q Li J Zhu A Long-lasting specific antibodies against P277 induced by mucosal administration of P277 repeat sequences carried by Hsp65 in the absence of adjuvants. Vaccine (2007) 25(11):2043–50. 10.1016/j.vaccine.2006.11.052 17224213

[18] Burkart V Germaschewski L Schloot NC Bellmann K Kolb H Deficient heat shock protein 70 response to stress in leukocytes at onset of type 1 diabetes. Biochem Biophys Res Commun (2008) 369(2):421–5. 10.1016/j.bbrc.2008.02.033 18282468

[19] Figueredo A Ibarra JL Rodriguez A Molino AM Gomez-de la Concha E Fernandez-Cruz A Increased serum levels of IgA antibodies to hsp70 protein in patients with diabetes mellitus: their relationship with vascular complications. Clin Immunol Immunopathol (1996) 79(3):252–5. 10.1006/clin.1996.0076 8635283

[20] Basu S Binder RJ Ramalingam T Srivastava PK CD91 is a common receptor for heat shock proteins gp96, hsp90, hsp70, and calreticulin. Immunity (2001) 14(3):303–13. 10.1016/S1074-7613(01)00111-X 11290339

[21] Delneste Y Magistrelli G Gauchat J Haeuw J Aubry J Nakamura K Involvement of LOX-1 in dendritic cell-mediated antigen cross-presentation. Immunity (2002) 17(3):353–62. 10.1016/S1074-7613(02)00388-6 12354387

[22] Fong JJ Sreedhara K Deng L Varki NM Angata T Liu Q Immunomodulatory activity of extracellular Hsp70 mediated via paired receptors Siglec-5 and Siglec-14. EMBO J (2015) 34(22):2775–88. 10.15252/embj.201591407 PMC4682649 26459514

[23] Qin HY Mahon JL Atkinson MA Chaturvedi P Lee-Chan E Singh B Type 1 diabetes alters anti-hsp90 autoantibody isotype. J Autoimmun (2003) 20(3):237–45. 10.1016/S0896-8411(03)00035-0 12753809

[24] Yilmaz A Gedikbasi A Yuruk Yildirim Z Pehlivanoglu C Seker B Sucu A Higher urine heat shock protein 70/creatinine ratio in type 1 diabetes mellitus. Ren Fail (2016) 38(3):404–10. 10.3109/0886022X.2015.1136893 26820050

[25] Takizawa S Endo T Wanjia X Tanaka S Takahashi M Kobayashi T HSP 10 is a new autoantigen in both autoimmune pancreatitis and fulminant type 1 diabetes. Biochem Biophys Res Commun (2009) 386(1):192–6. 10.1016/j.bbrc.2009.06.009 19520060

[26] Gruden G Bruno G Chaturvedi N Burt D Schalkwijk C Pinach S Serum heat shock protein 27 and diabetes complications in the EURODIAB prospective complications study: a novel circulating marker for diabetic neuropathy. Diabetes (2008) 57(7):1966–70. 10.2337/db08-0009 PMC2453614 18390793

[27] Lehmann PV Sercarz EE Forsthuber T Dayan CM Gammon G Determinant spreading and the dynamics of the autoimmune T-cell repertoire. Immunol Today (1993) 14(5):203–8. 10.1016/0167-5699(93)90163-F 7686009

[28] Marhfour I Lopez XM Lefkaditis D Salmon I Allagnat F Richardson SJ Expression of endoplasmic reticulum stress markers in the islets of patients with type 1 diabetes. Diabetologia (2012) 55(9):2417–20. 10.1007/s00125-012-2604-3 22699564

[29] Tersey SA Nishiki Y Templin AT Cabrera SM Stull ND Colvin SC Islet β-cell endoplasmic reticulum stress precedes the onset of type 1 diabetes in the nonobese diabetic mouse model. Diabetes (2012) 61(4):818–27. 10.2337/db11-1293 PMC3314371 22442300

[30] Engin F Yermalovich A Nguyen T Hummasti S Fu W Eizirik DL Restoration of the unfolded protein response in pancreatic β cells protects mice against type 1 diabetes. Sci Transl Med (2013) 5(211):211ra156. 10.1126/scitranslmed.3006534 PMC4169117 24225943

[31] Marré ML James EA Piganelli JD β cell ER stress and the implications for immunogenicity in type 1 diabetes. Front Cell Dev Biol (2015) 3:67. 10.3389/fcell.2015.00067 26579520 PMC4621612

[32] Eizirik DL Colli ML Ortis F The role of inflammation in insulitis and beta-cell loss in type 1 diabetes. Nat Rev Endocrinol (2009) 5(4):219–26. 10.1038/nrendo.2009.21 19352320

[33] Padgett LE Broniowska KA Hansen PA Corbett JA Tse HM The role of reactive oxygen species and proinflammatory cytokines in type 1 diabetes pathogenesis. Ann N Y Acad Sci (2013) 1281(1):16–35. 10.1111/j.1749-6632.2012.06826.x 23323860 PMC3715103

[34] Ursini F Maiorino M Forman HJ Redox homeostasis: The Golden Mean of healthy living. Redox Biol (2016) 8:205–15. 10.1016/j.redox.2016.01.010 PMC4732014 26820564

[35] Azevedo-Martins AK Lortz S Lenzen S Curi R Eizirik DL Tiedge M Improvement of the mitochondrial antioxidant defense status prevents cytokine-induced nuclear factor-kappaB activation in insulin-producing cells. Diabetes (2003) 52(1):93–101. 10.2337/diabetes.52.1.93 12502498

[36] Drews G Krippeit-Drews P Düfer M Oxidative stress and beta-cell dysfunction. Pflugers Arch (2010) 460(4):703–18. 10.1007/s00424-010-0862-9 20652307

[37] Curtsinger JM Schmidt CS Mondino A Lins DC Kedl RM Jenkins MK Inflammatory cytokines provide a third signal for activation of naive CD4+ and CD8+ T cells. J Immunol (1999) 162(6):3256–62. 10092777

[38] Lortz S Tiedge M Nachtwey T Karlsen AE Nerup J Lenzen S Protection of insulin-producing RINm5F cells against cytokine-mediated toxicity through overexpression of antioxidant enzymes. Diabetes (2000) 49(7):1123–30. 10.2337/diabetes.49.7.1123 10909968

[39] Coskun O Kanter M Korkmaz A Oter S Quercetin, a flavonoid antioxidant, prevents and protects streptozotocin-induced oxidative stress and beta-cell damage in rat pancreas. Pharmacol Res (2005) 51(2):117–23. 10.1016/j.phrs.2004.06.002 15629256

[40] Piganelli JD Flores SC Cruz C Koepp J Batinic-Haberle I Crapo J A metalloporphyrin-based superoxide dismutase mimic inhibits adoptive transfer of autoimmune diabetes by a diabetogenic T-cell clone. Diabetes (2002) 51(2):347–55. 10.2337/diabetes.51.2.347 11812741

[41] Feder ME Hofmann GE Heat-shock proteins, molecular chaperones, and the stress response: evolutionary and ecological physiology. Annu Rev Physiol (1999) 61:243–82. 10.1146/annurev.physiol.61.1.243 10099689

[42] Srivastava PK Peptide-binding heat shock proteins in the endoplasmic reticulum: role in immune response to cancer and in antigen presentation. Adv Cancer Res (1993) 62:153–77. 10.1016/S0065-230X(08)60318-8 8109317

[43] Todryk S Melcher AA Hardwick N Linardakis E Bateman A Colombo MP Heat shock protein 70 induced during tumor cell killing induces Th1 cytokines and targets immature dendritic cell precursors to enhance antigen uptake. J Immunol (1999) 163(3):1398–408. 10415040

[44] Murshid A Gong J Calderwood SK The role of heat shock proteins in antigen cross presentation. Front Immunol (2012) 3:63. 10.3389/fimmu.2012.00063 22566944 PMC3342350

[45] De Graeff-Meeder ER van der Zee R Rijkers GT Schuurman HJ Kuis W Bijlsma JW Recognition of human 60 kD heat shock protein by mononuclear cells from patients with juvenile chronic arthritis. Lancet (1991) 337(8754):1368–72. 10.1016/0140-6736(91)93057-G 1674762

[46] Bason C Corrocher R Lunardi C Puccetti P Olivieri O Girelli D Interaction of antibodies against cytomegalovirus with heat-shock protein 60 in pathogenesis of atherosclerosis. Lancet (2003) 362(9400):1971–7. 10.1016/S0140-6736(03)15016-7 14683657

[47] Elst EF Klein M de Jager W Kamphuis S Wedderburn LR van der Zee R Hsp60 in inflamed muscle tissue is the target of regulatory autoreactive T cells in patients with juvenile dermatomyositis. Arthritis Rheum (2008) 58(2):547–55. 10.1002/art.23202 18240224

[48] Pellegrino MW Nargund AM Haynes CM Signaling the mitochondrial unfolded protein response. Biochim Biophys Acta (2013) 1833(2):410–6. 10.1016/j.bbamcr.2012.02.019 PMC3393825 22445420

[49] Juwono J Martinus RD Does Hsp60 Provide a Link between Mitochondrial Stress and Inflammation in Diabetes Mellitus? J Diabetes Res (2016) 2016:8017571. 10.1155/2016/8017571 27478851 PMC4960334

[50] Quintana FJ Cohen IR The HSP60 immune system network. Trends Immunol (2011) 32(2):89–95. 10.1016/j.it.2010.11.001 21145789

[51] Birk OS Elias D Weiss AS Rosen A van-der Zee R Walker MD NOD mouse diabetes: the ubiquitous mouse hsp60 is a beta-cell target antigen of autoimmune T cells. J Autoimmun (1996) 9(2):159–66. 10.1006/jaut.1996.0019 8738959

[52] Brudzynski K Martinez V Gupta RS Secretory granule autoantigen in insulin-dependent diabetes mellitus is related to 62 kDa heat-shock protein (hsp60). J Autoimmun (1992) 5(4):453–63. 10.1016/0896-8411(92)90005-B 1418289

[53] Wick G Jakic B Buszko M Wick MC Grundtman C The role of heat shock proteins in atherosclerosis. Nat Rev Cardiol (2014) 11(9):516–29. 10.1038/nrcardio.2014.91 25027488

[54] Adler T Akiyama H Herder C Kolb H Burkart V Heat shock protein 60 elicits abnormal response in macrophages of diabetes-prone non-obese diabetic mice. Biochem Biophys Res Commun (2002) 294(3):592–6. 10.1016/S0006-291X(02)00522-3 12056808

[55] Sarikonda G Sachithanantham S Miller JF Pagni PP Coppieters KT von Herrath M The Hsp60 peptide p277 enhances anti-CD3 mediated diabetes remission in non-obese diabetic mice. J Autoimmun (2015) 59:61–6. 10.1016/j.jaut.2015.02.003 25772283

[56] Schloot NC Cohen IR DiaPep277® and immune intervention for treatment of type 1 diabetes. Clin Immunol (2013) 149(3):307–16. 10.1016/j.clim.2013.09.001 24090708

[57] Elias D Cohen IR Peptide therapy for diabetes in NOD mice. Lancet (1994) 343(8899):704–6. 10.1016/S0140-6736(94)91582-2 7907681

[58] Elias D Meilin A Ablamunits V Birk OS Carmi P Könen-Waisman S Hsp60 peptide therapy of NOD mouse diabetes induces a Th2 cytokine burst and downregulates autoimmunity to various beta-cell antigens. Diabetes (1997) 46(5):758–64. 10.2337/diabetes.46.5.758 9133541

[59] Ablamunits V Elias D Reshef T Cohen IR Islet T cells secreting IFN-gamma in NOD mouse diabetes: arrest by p277 peptide treatment. J Autoimmun (1998) 11(1):73–81. 10.1006/jaut.1997.0177 9480725

[60] Abulafia-Lapid R Elias D Raz I Keren-Zur Y Atlan H Cohen IR T cell proliferative responses of type 1 diabetes patients and healthy individuals to human hsp60 and its peptides. J Autoimmun (1999) 12(2):121–9. 10.1006/jaut.1998.0262 10047432

[61] Nussbaum G Zanin-Zhorov A Quintana F Lider O Cohen IR Peptide p277 of HSP60 signals T cells: inhibition of inflammatory chemotaxis. Int Immunol (2006) 18(10):1413–9. 10.1093/intimm/dxl074 16893923

[62] Raz I Elias D Avron A Tamir M Metzger M Cohen IR Beta-cell function in new-onset type 1 diabetes and immunomodulation with a heat-shock protein peptide (DiaPep277): a randomised, double-blind, phase II trial. Lancet (2001) 358(9295):1749–53. 10.1016/S0140-6736(01)06801-5 11734230

[63] Schloot NC Meierhoff G Lengyel C Vandorfi G Takacs J Panczel P Effect of heat shock protein peptide DiaPep277 on beta-cell function in paediatric and adult patients with recent-onset diabetes mellitus type 1: two prospective, randomized, double-blind phase II trials. Diabetes Metab Res Rev (2007) 23(4):276–85. 10.1002/dmrr.707 17103487

[64] Huurman VA Decochez K Mathieu C Cohen IR Roep BO Therapy with the hsp60 peptide DiaPep277 in C-peptide positive type 1 diabetes patients. Diabetes Metab Res Rev (2007) 23(4):269–75. 10.1002/dmrr.691 17024692

[65] Lazar L Ofan R Weintrob N Avron A Tamir M Elias D Heat-shock protein peptide DiaPep277 treatment in children with newly diagnosed type 1 diabetes: a randomised, double-blind phase II study. Diabetes Metab Res Rev (2007) 23(4):286–91. 10.1002/dmrr.711 17124721

[66] Huurman VA van der Meide PE Duinkerken G Willemen S Cohen IR Elias D Immunological efficacy of heat shock protein 60 peptide DiaPep277 therapy in clinical type I diabetes. Clin Exp Immunol (2008) 152(3):488–97. 10.1111/j.1365-2249.2008.03656.x PMC2453210 18422727

[67] Tukaj S Kaminski M Heat shock proteins in the therapy of autoimmune diseases: too simple to be true? Cell Stress Chaperones (2019) 24(3):475–9. 10.1007/s12192-019-01000-3 PMC6527538 31073900

[68] Gaofu Q Dan M Jie W Liao Z Li Z Roque RS Long-lasting specific antibodies against CETP induced by subcutaneous and mucosal administration of a 26-amino acid CETP epitope carried by heat shock protein 65 kDa in the absence of adjuvants. Vaccine (2004) 22(23-24):3187–94. 10.1016/j.vaccine.2004.01.060 15297073

[69] Jindal S Dudani AK Singh B Harley CB Gupta RS Primary structure of a human mitochondrial protein homologous to the bacterial and plant chaperonins and to the 65-kilodalton mycobacterial antigen. Mol Cell Biol (1989) 9(5):2279–83. 10.1128/MCB.9.5.2279 PMC363030 2568584

[70] Elias D Markovits D Reshef T van der Zee R Cohen IR Induction and therapy of autoimmune diabetes in the non-obese diabetic (NOD/Lt) mouse by a 65-kDa heat shock protein. Proc Natl Acad Sci USA (1990) 87(4):1576–80. 10.1073/pnas.87.4.1576 PMC53518 2406723

[71] Jones DB Hunter NR Duff GW Heat-shock protein 65 as a beta cell antigen of insulin-dependent diabetes. Lancet (1990) 336(8715):583–5. 10.1016/0140-6736(90)93390-B 1975377

[72] Tun RY Smith MD Lo SS Rook GA Lydyard P Leslie RD Antibodies to heat shock protein 65 kD in type 1 diabetes mellitus. Diabetes Med (1994) 11(1):66–70. 10.1111/j.1464-5491.1994.tb00232.x 8181256

[73] Lu S Li G Liu K Yang X Cao R Zong L Fusion protein His-Hsp65-6IA2P2 prevents type 1 diabetes through nasal immunization in NOD Mice. Int Immunopharmacol (2016) 35:235–42. 10.1016/j.intimp.2016.03.024 27082999

[74] Van Eden W Wick G Albani S Cohen I Stress, heat shock proteins, and autoimmunity: how immune responses to heat shock proteins are to be used for the control of chronic inflammatory diseases. Ann N Y Acad Sci (2007) 1113:217–37. 10.1196/annals.1391.020 17584980

[75] Tukaj S Heat Shock Protein 70 as a Double Agent Acting Inside and Outside the Cell: Insights into Autoimmunity. Int J Mol Sci (2020) 21(15):5298. 10.3390/ijms21155298 PMC7432326 32722570

[76] Eizirik DL Welsh M Strandell E Welsh N Sandler S Interleukin-1 beta depletes insulin messenger ribonucleic acid and increases the heat shock protein hsp70 in mouse pancreatic islets without impairing the glucose metabolism. Endocrinology (1990) 127(5):2290–7. 10.1210/endo-127-5-2290 2171911

[77] Bellmann K Wenz A Radons J Burkart V Kleemann R Kolb H Heat shock induces resistance in rat pancreatic islet cells against nitric oxide, oxygen radicals and streptozotocin toxicity in vitro. J Clin Investigation (1995) 95(6):2840–5. 10.1172/JCI117989 PMC295970 7769124

[78] Burkart V Liu H Bellmann K Wissing D Jaattela M Cavallo MG Natural resistance of human beta cells toward nitric oxide is mediated by heat shock protein 70. J Biol Chem (2000) 275(26):19521–8. 10.1074/jbc.M002265200 10751413

[79] Kumar GS Kulkarni A Khurana A Kaur J Tikoo K Selenium nanoparticles involve HSP-70 and SIRT1 in preventing the progression of type 1 diabetic nephropathy. Chem Biol Interact (2014) 223:125–33. 10.1016/j.cbi.2014.09.017 25301743

[80] Bellmann K Hui L Radons J Burkart V Kolb H Low stress response enhances vulnerability of islet cells in diabetes-prone BB rats. Diabetes (1997) 46(2):232–6. 10.2337/diabetes.46.2.232 9000699

[81] Wachlin G Heine L Klöting I Dunger A Hahn HJ Schmidt S Stress response of pancreatic islets from diabetes prone BB rats of different age. Autoimmunity (2002) 35(6):389–95. 10.1080/0891693021000014989 12568119

[82] Gerazova-Efremova K Dinevska-Kjovkarovska S Miova B Heat-Shock Protein 70-Mediated Heat Preconditioning Attenuates Hepatic Carbohydrate and Oxidative Disturbances in Rats With Type 1 Diabetes. Can J Diabetes (2019) 43(5):345–53. 10.1016/j.jcjd.2019.01.002 30853267

[83] Binder RJ Functions of heat shock proteins in pathways of the innate and adaptive immune system. J Immunol (2014) 193(12):5765–71. 10.4049/jimmunol.1401417 PMC4304677 25480955

[84] Burkart V Siegenthaler RK Blasius E Vandenbroeck K Alloza I Fingberg W High affinity binding of hydrophobic and autoantigenic regions of proinsulin to the 70 kDa chaperone DnaK. BMC Biochem (2010) 11:44. 10.1186/1471-2091-11-44 21059249 PMC2994776

[85] Krause M Heck TG Bittencourt A Scomazzon SP Newsholme P Curi R The chaperone balance hypothesis: the importance of the extracellular to intracellular HSP70 ratio to inflammation-driven type 2 diabetes, the effect of exercise, and the implications for clinical management. Mediators Inflammation (2015) 2015:249205. 10.1155/2015/249205 PMC4357135 25814786

[86] Vomund AN Zinselmeyer BH Hughes J Calderon B Valderrama C Ferris ST Beta cells transfer vesicles containing insulin to phagocytes for presentation to T cells. Proc Natl Acad Sci USA (2015) 112(40):E5496–502. 10.1073/pnas.1515954112 PMC4603448 26324934

[87] Cianciaruso C Phelps EA Pasquier M Hamelin R Demurtas D Alibashe Ahmed M Primary Human and Rat β-Cells Release the Intracellular Autoantigens GAD65, IA-2, and Proinsulin in Exosomes Together With Cytokine-Induced Enhancers of Immunity. Diabetes (2017) 66(2):460–73. 10.2337/db16-0671 27872147

[88] Lancaster GI Febbraio MA Exosome-dependent trafficking of HSP70: a novel secretory pathway for cellular stress proteins. J Biol Chem (2005) 280(24):23349–55. 10.1074/jbc.M502017200 15826944

[89] Tamura Y Yoneda A Takei N Sawada K Spatiotemporal Regulation of Hsp90-Ligand Complex Leads to Immune Activation. Front Immunol (2016) 7:201. 10.3389/fimmu.2016.00201 27252703 PMC4877505

[90] Watkins RA Evans-Molina C Terrell JK Day KH Guindon L Restrepo IA Proinsulin and heat shock protein 90 as biomarkers of beta-cell stress in the early period after onset of type 1 diabetes. Transl Res (2016) 168:96–106.e1. 10.1016/j.trsl.2015.08.010 26397425 PMC4839287

[91] Ocaña GJ Sims EK Watkins RA Ragg S Mather KJ Oram RA Analysis of serum Hsp90 as a potential biomarker of β cell autoimmunity in type 1 diabetes. PLoS One (2019) 14(1):e0208456. 10.1371/journal.pone.0208456 30629603 PMC6328179

[92] Novoselova EG Glushkova OV Khrenov MO Parfenyuk SB Lunin SM Novoselova TV Participation of Hsp70 and Hsp90α Heat Shock Proteins in Stress Response in the Course of Type 1 Diabetes Mellitus. Dokl Biol Sci (2020) 493(1):124–7. 10.1134/S0012496620040079 32894426

[93] Houlihan JL Metzler JJ Blum JS HSP90alpha and HSP90beta isoforms selectively modulate MHC class II antigen presentation in B cells. J Immunol (2009) 182(12):7451–8. 10.4049/jimmunol.0804296 PMC2714911 19494268

[94] Clayton A Turkes A Navabi H Mason MD Tabi Z Induction of heat shock proteins in B-cell exosomes. J Cell Sci (2005) 118(Pt 16):3631–8. 10.1242/jcs.02494 16046478

[95] Bradfield JP Qu HQ Wang K Zhang H Sleiman PM Kim CE A genome-wide meta-analysis of six type 1 diabetes cohorts identifies multiple associated loci. PLoS Genet (2011) 7(9):e1002293. 10.1371/journal.pgen.1002293 21980299 PMC3183083

[96] Ladiges WC Knoblaugh SE Morton JF Korth MJ Sopher BL Baskin CR Pancreatic beta-cell failure and diabetes in mice with a deletion mutation of the endoplasmic reticulum molecular chaperone gene P58IPK. Diabetes (2005) 54(4):1074–81. 10.2337/diabetes.54.4.1074 15793246

[97] Synofzik M Haack TB Kopajtich R Gorza M Rapaport D Greiner M Absence of BiP co-chaperone DNAJC3 causes diabetes mellitus and multisystemic neurodegeneration. Am J Hum Genet (2014) 95(6):689–97. 10.1016/j.ajhg.2014.10.013 PMC4259973 25466870

[98] Vig S Buitinga M Rondas D Crèvecoeur I van Zandvoort M Waelkens E Cytokine-induced translocation of GRP78 to the plasma membrane triggers a pro-apoptotic feedback loop in pancreatic beta cells. Cell Death Dis (2019) 10(4):309. 10.1038/s41419-019-1518-0 30952835 PMC6450900

[99] Wiersma VR Michalak M Abdullah TM Bremer E Eggleton P Mechanisms of Translocation of ER Chaperones to the Cell Surface and Immunomodulatory Roles in Cancer and Autoimmunity. Front Oncol (2015) 5:7. 10.3389/fonc.2015.00007 25688334 PMC4310273

[100] Athanasas-Platsis S Zhang B Hillyard NC Cavanagh AC Csurhes PA Morton H Early pregnancy factor suppresses the infiltration of lymphocytes and macrophages in the spinal cord of rats during experimental autoimmune encephalomyelitis but has no effect on apoptosis. J Neurol Sci (2003) 214(1-2):27–36. 10.1016/S0022-510X(03)00170-9 12972385

[101] Zanin-Zhorov A Tal G Shivtiel S Cohen M Lapidot T Nussbaum G Heat shock protein 60 activates cytokine-associated negative regulator suppressor of cytokine signaling 3 in T cells: effects on signaling, chemotaxis, and inflammation. J Immunol (2005) 175(1):276–85. 10.4049/jimmunol.175.1.276 15972659

[102] Brugman S Klatter FA Visser J Bos NA Elias D Rozing J Neonatal oral administration of DiaPep277, combined with hydrolysed casein diet, protects against Type 1 diabetes in BB-DP rats. An experimental study. Diabetologia (2004) 47(7):1331–3. 10.1007/s00125-004-1452-1 15248047

[103] Quintana FJ Carmi P Cohen IR DNA vaccination with heat shock protein 60 inhibits cyclophosphamide-accelerated diabetes. J Immunol (2002) 169(10):6030–5. 10.4049/jimmunol.169.10.6030 12421990

[104] El-Horany HE Abd-Ellatif RN Watany M Hafez YM Okda HI NLRP3 expression and urinary HSP72 in relation to biomarkers of inflammation and oxidative stress in diabetic nephropathy patients. IUBMB Life (2017) 69(8):623–30. 10.1002/iub.1645 28631886

[105] Morteza A Nakhjavani M Larry M Nargesi AA Esteghamati A Heat shock protein 70 and albuminuria in patients with type 2 diabetes: a matched case control study. Cell Stress Chaperones (2013) 18(6):815–9. 10.1007/s12192-013-0435-x PMC3789871 23681922

[106] Hooper PL Tytell M Vígh L Xenohormesis: health benefits from an eon of plant stress response evolution. Cell Stress Chaperones (2010) 15(6):761–70. 10.1007/s12192-010-0206-x PMC3024065 20524162

[107] Padmalayam I Targeting mitochondrial oxidative stress through lipoic acid synthase: a novel strategy to manage diabetic cardiovascular disease. Cardiovasc Hematol Agents Med Chem (2012) 10(3):223–33. 10.2174/187152512802651060 22632266

[108] Oksala NK Lappalainen J Laaksonen DE Khanna S Kaarniranta K Sen CK Alpha-lipoic Acid modulates heat shock factor-1 expression in streptozotocin-induced diabetic rat kidney. Antioxid Redox Signal (2007) 9(4):497–506. 10.1089/ars.2006.1450 17280490

[109] Zhang HM Dang H Kamat A Yeh CK Zhang BX Geldanamycin derivative ameliorates high fat diet-induced renal failure in diabetes. PLoS One (2012) 7(3):e32746. 10.1371/journal.pone.0032746 22412919 PMC3295767

[110] Cheng CF Sahu D Tsen F Zhao Z Fan J Kim R A fragment of secreted Hsp90α carries properties that enable it to accelerate effectively both acute and diabetic wound healing in mice. J Clin Invest (2011) 121(11):4348–61. 10.1172/JCI46475 PMC3204835 22019588

[111] Verrijn Stuart AA de Jager W Klein MR Teklenburg G Nuboer R Hoorweg JJ Recognition of heat shock protein 60 epitopes in children with type 1 diabetes. Diabetes Metab Res Rev (2012) 28(6):527–34. 10.1002/dmrr.2306 22492505

[112] Sargent CA Dunham I Trowsdale J Campbell RD Human major histocompatibility complex contains genes for the major heat shock protein HSP70. Proc Natl Acad Sci U S A (1989) 86(6):1968–72. 10.1073/pnas.86.6.1968 PMC286826 2538825

[113] Wurst W Benesch C Drabent B Rothermel E Benecke BJ Günther E Localization of heat shock protein 70 genes inside the rat major histocompatibility complex close to class III genes. Immunogenetics (1989) 30(1):46–9. 10.1007/BF02421469 2568336

[114] Gaskins HR Prochazka M Nadeau JH Henson VW Leiter EH Localization of a mouse heat shock Hsp70 gene within the H-2 complex. Immunogenetics (1990) 32(4):286–9. 10.1007/BF00187100 1978715

[115] Brorsson C Hansen NT Lage K Bergholdt R Brunak S Pociot F Identification of T1D susceptibility genes within the MHC region by combining protein interaction networks and SNP genotyping data. Diabetes Obes Metab (2009) 11(Suppl 1Suppl 1):60–6. 10.1111/j.1463-1326.2008.01004.x PMC2755052 19143816

[116] Pociot F Rønningen KS Nerup J Polymorphic analysis of the human MHC-linked heat shock protein 70 (HSP70-2) and HSP70-Hom genes in insulin-dependent diabetes mellitus (IDDM). Scand J Immunol (1993) 38(5):491–5. 10.1111/j.1365-3083.1993.tb02593.x 7901896

[117] De Maio A Extracellular heat shock proteins, cellular export vesicles, and the Stress Observation System: a form of communication during injury, infection, and cell damage. It is never known how far a controversial finding will go! Dedicated to Ferruccio Ritossa. Cell Stress Chaperones (2011) 16(3):235–49. 10.1007/s12192-010-0236-4 PMC3077223 20963644

[118] Chow AM Brown IR Induction of heat shock proteins in differentiated human and rodent neurons by celastrol. Cell Stress Chaperones (2007) 12(3):237–44. 10.1379/CSC-269.1 PMC1971233 17915556

[119] Rawshani A Sattar N Franzén S Hattersley AT Svensson AM Eliasson B Excess mortality and cardiovascular disease in young adults with type 1 diabetes in relation to age at onset: a nationwide, register-based cohort study. Lancet (2018) 392(10146):477–86. 10.1016/S0140-6736(18)31506-X PMC6828554 30129464

